# Medlay: A Reconfigurable Micro-Power Management to Investigate Self-Powered Systems

**DOI:** 10.3390/s18010259

**Published:** 2018-01-17

**Authors:** Jan Kokert, Tobias Beckedahl, Leonhard M. Reindl

**Affiliations:** Laboratory for Electrical Instrumentation, Department of Microsystems Engineering—IMTEK, University of Freiburg, Georges-Köhler-Allee 106, 79110 Freiburg, Germany; becksed@googlemail.com (T.B.); reindl@imtek.uni-freiburg.de (L.M.R.)

**Keywords:** Energy harvesting, Power management, Smart sensors, System analysis and design

## Abstract

In self-powered microsystems, a power management is essential to extract, transfer and regulate power from energy harvesting sources to loads such as sensors. The challenge is to consider all of the different structures and components available and build the optimal power management on a microscale. The purpose of this paper is to streamline the design process by creating a novel reconfigurable testbed called Medlay. First, we propose a uniform interface for management functions e.g., power conversion, energy storing and power routing. This interface results in a clear layout because power and status pins are strictly separated, and inputs and outputs have fixed positions. Medlay is the ready-to-use and open-hardware platform based on the interface. It consists of a base board and small modules incorporating e.g., dc-dc converters, power switches and supercapacitors. Measurements confirm that Medlay represents a system on one circuit board, as parasitic effects of the interconnections are negligible. The versatility regarding different setups on the testbed is determined to over 250,000 combinations by layout graph grammar. Lastly, we underline the applicability by recreating three state-of-the-art systems with the testbed. In conclusion, Medlay facilitates building and testing power management in a very compact, clear and extensible fashion.

## 1. Introduction

Wireless sensor nodes (WSN) are electrical micro-systems which continuously measure physical parameters in their surrounding. The readings are e.g., temperature, pressure, humidity [1], light spectrum [2], vibration of critical infrastructure [3], soil properties [4] or pavement traffic [5]. This data is then transferred by the node to the next node or to a central unit for further processing.

### 1.1. Energy Harvesting Powered Sensor Systems

To supply power to autonomous operating WSNs, energy harvesting (EH) is one solution of choice [6]. EH utilizes the energy of the surroundings (no matter in which form) and converts it into electrical energy. The energy amounts here are very small compared to typical power systems and range from 10 μW to 100 mW [6]. Nevertheless, this is enough to supply power to microsystems such as a low-power microcontroller, a sensor and a radio frontend (RF). All of these loads need a constant regulated voltage. The current instead is pulsing as the loads are switched off as often as possible to save energy which is called duty-cycling. Furthermore, the current profile is determined by the application because the node has to measure and transfer data periodically. This is contrary to the energy harvested, which fluctuates due to the environmental conditions [7]. Consequently, the instantaneous power is unbalanced, which would lead to an problem if the energy harvester was directly conected to the system load. This problem is usually mitigated by an intermediate energy storage [8]; however, it leads to more challenges regarding a proper charge management. A further issue during system design is that the voltage level of the harvester does not match the voltage of a typical load e.g., a microcontroller with 3.3 V. Typical harvester voltages are 0.5 V for a single solar cell and >10 V for a piezoelectric harvester [9,10].

**Figure 1:**
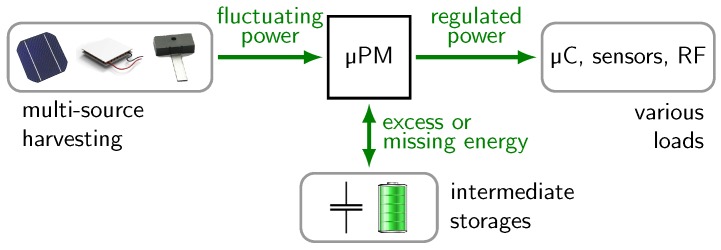
The micro-power management (µPM) supplies regulated power to a load from energy harvesting sources.

All mentioned issues are addressed with a micro-power management (µPM). The µPM is in-between the harvesters, the storages and the loads and is essential to extract, transfer and regulate power. This basic setup is illustrated in Figure 1. For comparison, mid-power systems in the field of renewable energies and microgrid show similar functional requirements. However, the design and the components are very different because typical powers are in the range from 100 W to 10 kW [11,12] which is about six orders of magnitude greater than the powers of energy-harvesting systems.

### 1.2. Detailed Structure of a Power Management

**Figure 2:**
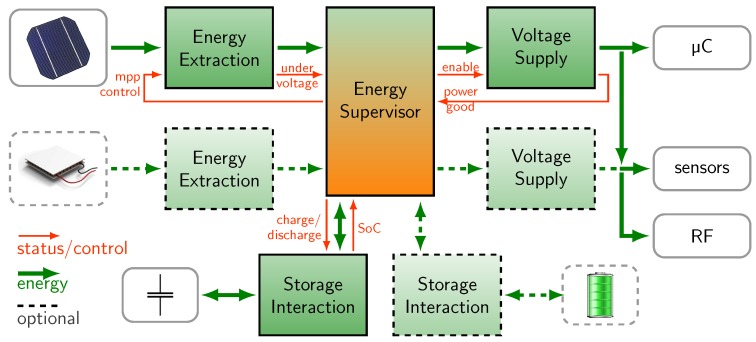
An illustration of a function-oriented structure of a power management. In a real system the functions *energy extraction*, *storage interaction* and *voltage supply* shown in green are realized with power converters.

In Figure 2 a more detailed structure of a power management is presented. We structured the system that basic functions of a µPM are represented by *functional blocks*, which are shown with a black rectangular outline. To realize advanced features like multi-harvesting or a hybrid storage, more functional blocks may be added which is illustrated with a dashed line.

The flow of energy shown in green is clearly separated from control signals shown in orange. There are other representations of power management structures in literature, but they are too vague and not standardized like [13]. The purpose of the green functional blocks, which only handle energy, is explained in the following:

**Energy Extraction** is required to extract as much energy from a harvester as possible. The power supplied by a harvester (e.g., a solar cell) depends on the environmental power (e.g., illumination) and its voltage operating point. For each input power there is one operating point (the maximum power point, MPP) at which the extracted power is at maximum. As the environmental conditions change, the MPP needs to be tracked continuously by controlling the terminal voltage of the harvester. For MPP tracking (MPPT) different methods can be used, such as the fractional open-circuit voltage method (FOCV) [14], perturb and observe (P&O) method [15], fuzzy logic [12] or a neural network [16]. For mid-power systems the main focus is to optimize the conversion efficiency and there is enough energy available to power a fuzzy logic or a neural network. For EH-systems the main focus is on reducing standby power and quiescent currents and only the low-power FOCV and P&O methods are appropriate.

**Voltage Supply** is required to supply a constant voltage to a load (e.g., 3.3 V a microcontroller and a sensor). Here, a power converter is used with a feedback configuration which regulates the output voltage. Commonly, this is realized by a resistor divider between the output pin and ground. The mid tap is then fed to an error amplifier.

**Storage Interaction** is needed to charge and discharge energy storages properly and prevent under or over-charging. Most secondary batteries (Li-ion, LiPo) need a constant-current, constant-voltage charging scheme (CCCV). Due to power fluctuations this scheme cannot be followed completely, but at least voltage limiting for under and over-charge protection must be implemented. The feedback configuration of the converter is then a combination of controlling the output current and output voltage.

The **Energy Supervisor** handles energy and control signals which are interwoven and thus illustrated by green and orange merging into one another. The energy supervisor covers all higher-order functions of a power management and guaranties the interplay of the aforementioned blocks. Energy supervision functions (here simplified as only one block) can be used for the following tasks: (1) to sense internal states, (2) to process data and (3) to control power. In practice, the following devices are used: current and voltage sense circuits, or-ing diodes and load switches. Common names for control signals are *chip enable*, *power good*, and *undervoltage lockout*. More examples are listed in Table 1, where the structure is applied to six state-of-the-art systems.

### 1.3. Challenge: Realization of a µPM Using PMICs

In the function-oriented structure in Figure 2, power converters (the green blocks) only have the purpose to transform voltage and current from one level to another. The term power converter comprises of dc-dc converters, charge pumps and low drop-out regulators (LDOs). They are used to realize the three mentioned µPM functions (Energy Extraction, Voltage Supply, and Storage Interaction) by means of different closed-loop feedback configurations. The whole µPM hardware (all green and orange blocks in Figure 2) is realized either with custom circuitry or with power management integrated circuits (PMICs). The term PMIC refers to a wide range of chips commercially available for electric power conversion or energy supervision or a combination of both.

State-of-the-art dc-dc converters, such as the BQ25570 from Texas Instruments, integrate more functions in one IC than just the power conversion. This IC offers MPPT via FOCV, where every 18 s the open-circuit voltage is sampled and scaled by a resistor divider. Furthermore, a voltage comparator and a load switch are implemented. All these functions Functions which belong structurally to the energy supervisor. Revising the structure in Figure 2, the focus on power converters without supervising functionality leads to a good system understanding. However, the structure cannot represent the use of PMICs. This issue will be addressed in Section 3.1. Our solution (general hardware blocks), and the implementation as a reconfigurable testbed is presented in Section 3.3.

### 1.4. Research Question

Considering the structure in Figure 2 and the variety of PMIC available in the context of development and prototyping, following key question arises: *“Is there a general physical structure for micro-power management which is flexible enough to set-up various configurations easily?”*

The question is relevant as there is a huge variety of how to realize a micro-power management. This variety starts with the structure of the management and is followed by the implementation using discrete electronic components and PMICs. Regarding PMICs, numerous pins need to be connected correctly; external components like inductors, need to be selected carefully; and layout constrains need to be followed. All of this non-standardized work can be very cumbersome for the designer and distracts from the overall system design. Furthermore, there are numerous storage technologies available such as: electric double-layer capacitor (EDLC, short: supercaps), thin-film batteries and various types of secondary batteries. Therefore a researcher, a design engineer or a student faces the challenging task of designing the optimal power management. Furthermore, an *optimal* solution is subjective and may mean the cheapest or the smallest or the most robust or the most energy-efficient solution. The motivation and application of our work are WSN. However, the concept and the reconfigurable testbed can be used during the design of any embedded system, preferably of self-powered ones.

The remainder of the paper is organized as follows: First we present in Section 2 related work. Then we present our solution concept in detail within Section 3. Afterwards, in Section 4 we show the influence of parasitic effects and demonstrate the versatility of the testbed. Finally, we discuss our solution with respect to other modular platforms in Section 5, and conclude the paper in Section 6.

## 2. Related Work

Within this section advanced µPM concepts in literature are presented. This documents the various components and architectures which are possible when designing a self-powered WSN. For a better overview all systems are summarized in Table 1. Afterwards, commercial and scientific platforms are presented which are designed for testing and prototyping. How our system outperforms state-of-the-art modular systems and sets new standards is shown in the discussion section.

### 2.1. Advanced Micro-Power Managements

Reference [17] shows that using a dc-dc converter and analog switches prolong the life of battery-powered WSNs by 30%. To achieve the high system efficiency, dynamic voltage scaling for different application tasks is applied and three power switches to override the dc-dc converter in certain situations are used. This observation complies with the fact, that all the following designs use dc-dc converters to increase the system efficiency:

**Ambimax** presented in [18], utilizes wind and solar harvesting to power a WSN. The energy is extracted by two LTC3401 dc-dc converter from each harvester. The maximum power point is tracked by a pilot element (a light sensor and a speed sensor, respectively) which controls the dc-dc converter utilizing a comparator. Energy from these stages are buffered by super capacitors and merged using diodes. Furthermore, a charge/discharge function for a LiPo battery is realized, utilizing a current-limiting switch and comparators. The control is entirely analog without involving a microcontroller.

**Trio** documented in [19] is a WSN prototype board powered by ten miniature photovoltaic (PV) cells and the BQ25504 dc-dc converter. The control of the power management is implemented in a complex programmable logic device (CPLD). The built-in MPPT (FOCV) of the BQ25504 is not used, but instead controlled by a pilot cell and the CPLD. Internal voltages and currents are sampled using a four-channel analog-to-digital converter (ADC). The authors realized a hybrid storage with a super capacitor and a Li-ion battery. A custom H-bridge bidirectional buck-boost converter was built to transfer energy between the storages.

**The Hybrid Storage System** described in [20] proposes a central 3.3 V dc-bus, which is accessed by bidirectional dc-dc converters. The dc-dc converters are built using the TPS43000 dc-dc controller and external MOS transistors. Thus, a hybrid storage comprising of a super capacitor and a Li-ion battery is realized. The authors used a microcontroller for energy supervision to control the charging direction between the storages depending on their voltage.

**Wang et al.** document in [21] the design of a WSN which is powered by a thermoelectric generator (TEG). The purpose of the WSN is to measure temperature, humidity, illumination and motion in buildings. Energy from the TEG is extracted by a two-stage concept utilizing a Seiko charge pump and the TPS61020 boost converter. The intermediate storage is a supercap array. A regulated output voltage for the load is provided by a TPS61220 boost converter.

**Li et al.** present in [22] a system for multisource harvesting from vibration and solar energy sources. Similar to Ambimax, the LTC3401 converter is used together with the LT1440 comparator. For accessing the piezo harvester, a rectifier and a step-down converter (LTC3632) are used. Both energy outputs are merged using Schottky diodes to charge a 25 mF supercap. Excess energy is transferred to a LiPo battery which is controlled by a pMOS transistor and a resistor divider. To increase the usable energy from the supercap, a buck-boost converter is used to supply a regulated output voltage.

**InfiniTime** published in [23] is an intelligent wristband powered by mini TEGs and PV cells. State-of-the-art power converters (LT3108 and BQ25570) are used to extract energy. The harvested energies are merged by diodes (power or-ing) and stored on a small LiPo battery. A constant voltage for the wristband electronics is provided by using the additional dc-dc buck converter of the BQ25570.

### 2.2. Platforms for Testing and Prototyping

**The modular plug-and-play platform** presented in [24] is based on a multiplexer module. Several energy components, such as a supercap, a battery or a PV module, are connected as boards via RJ45 cables. The design aims for a system which can be changed *during* runtime. Thus, each energy module includes a suitable dc-dc converter, protection circuitry and an EEPROM (electrically erasable programmable read-only memory) with electrical data. A microcontroller interrogates the modules using the multiplexer to control states and read out voltage levels.

**Bader et al.** show in [25] a configurable energy harvesting platform for evaluation of short-term energy storages. Supercaps and thin-film batteries are compared in fixed combination with a step-up regulator and an LDO. For energy extraction, an additional dc-dc converter (LT3105) can be activated.

**Prototyping boards** mentioned in [26] form a modular platform to evaluate different dc-dc converters and loads. Each component of a power management is routed on a uniform board (10×16 cm). The boards are connected side-by-side with 32-pin connectors to form one wide bus. The assertion of the 32 input/output (I/O) lines is totally free - each module picks the relevant traces by a 3.2${}^{\prime \prime }$ × 3.2${}^{\prime \prime }$patch field.

Considering **commercial evaluation boards** for PMIC, one will notice that through all well-known manufacturers the boards are not standardized by any means. However, some companies offer evaluation kits for *their* PMICs including a solar cell and an energy storage. Examples are the *ADP5091* demo board from *Analog Devices* [27], the *Maxim Integrated MAX17710 evaluation board*, the *enOcean EVA320-2 board* and the evaluation board called *IPS-EVAL-EH-01* from *Infinite Power Solutions*. In the following, three high-advanced evaluation boards are presented: The German company *Würth-Elektronik* offers a commercial kit called *Energy Harvesting Solution to go* [28]. The kit includes two harvesters (a solar cell and a TEG) and four different dc-dc converters from Linear Technology on one PCB (printed circuit board). The individual functions can be chosen by jumpers. As a load, an EFM32 Giant Gecko Starter Kit from Silicon Labs can be connected. The successor-board called *Gleanergy* [29] focuses on ICs offering energy backup with primary batteries. The load was advanced to a smartMesh-IP WSN with E Ink display. Again, the circuitry of interest is enabled by jumpers.

Actually, the most modular commercial solution is the *Energy Harvesting Platform* from *Future Electronics* [30]. Different harvesters, dc-dc converters, storages and loads can be connected via a uniform interface to one base platform. However, the structure is limited to a maximum of one source module, two dc-dc converter modules, one storage module and one application module (load). Furthermore, the base platform offers bi-stable relays for power routing and current measurement.

## 3. Methods and Materials

Our solution concept and the implementation is guided by the following design aspects:

**Think in a top-level structure** of the power management using individual building blocks. Apply a *divide and conquer* approach, which helps to focus on the main structure of the system, whereas starting with layout and component considerations first can be distractive and time-consuming.

**Combine building blocks** to a complete power management like constructing with LEGO^®^. The interface of the blocks must be clear and any influence on the system due interconnections must be as low as possible. Circuit parts which are not used in one system shall be removed physically to keep the setup clear. Jumpers or even multiplexers shall not be used for that as they unnecessarily increase the complexity.

**Plug and play experience** for the developer when testing and optimizing the system, e.g., when selectively repairing a sub-circuit or when comparing different configurations. For error tracing and measurement tasks the interconnections between blocks shall be interruptible.

### 3.1. Our Concept—General Hardware Blocks

The challenge is to build a system in a general homogeneous fashion based on PMIC (power management integrated circuits) whereas PMICs themselves offer multiple heterogenous functions. For clarification, we analyzed commercially available PMIC regarding their number of inputs and outputs for power and control. The results are shown in Table 2 which summarizes the pin assignments of components grouped by different types. The pin assignment of the vast majority of PMIC fit into the table. A rare exception is the LT3108 for example, which offers three power outputs (V${}_{\mathrm{STORE}}$, V${}_{\mathrm{OUT}2}$, V${}_{\mathrm{LDO}}$) but only two can be chosen as power output.

We identify a basic element namely the *general hardware block* which occurs nine times in Figure 2 and follows three assumptions:

(1) Every general block is capable of handling both energy and control signals. Nevertheless, the domains remain physically separated in the actual implementation (see Section 3.3).
(2) Every general block has an interface of up to two power inputs and up to two power outputs. Furthermore, the interface includes up to four control inputs and up to four control outputs.
(3) Energy storages are considered as general blocks like power converters. They are not external, as shown in Figure 1 but are part of the power management.

**Figure 3:**
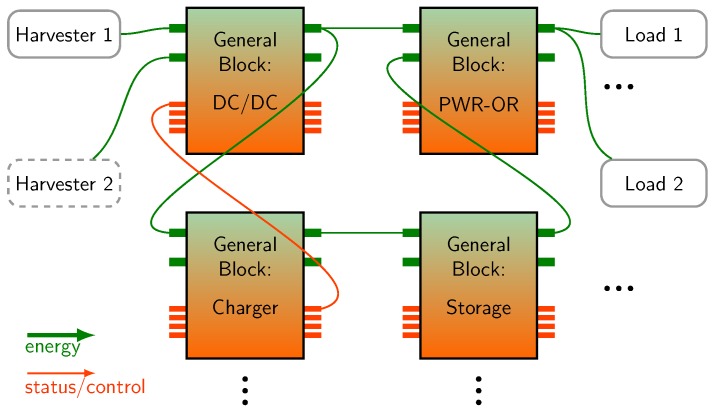
A homogeneous structure of a power management built up from general hardware blocks with fixed interface for energy and control.

In Figure 3 an example with four general blocks is illustrated. The interfaces of the blocks (inputs and outputs for power and control) are highlighted with thicker lines.

### 3.2. The implementation—From General Blocks to Modules

Based on the general hardware blocks we created a modular platform called Medlay. Here, we put emphasis on power traces of the µPM for two reasons: (1) power traces represent the system architecture and should be laid out clearly. (2) power traces are responsible for ohmic losses and should be kept as short as possible. Furthermore, energy blocks are usually chained successively, e.g., the output of block 1 is connected to the input of block 2. In contrast to that, control and status signals do not follow any particular pattern and do not require a low-ohmic connection.

Our result of focusing on the power path is a base board with low-ohmic power traces and exchangeable uniform modules. Each module implements one general hardware block. The details are explained in the next two sections.

### 3.3. Standard Module Interface

**Figure 4:**
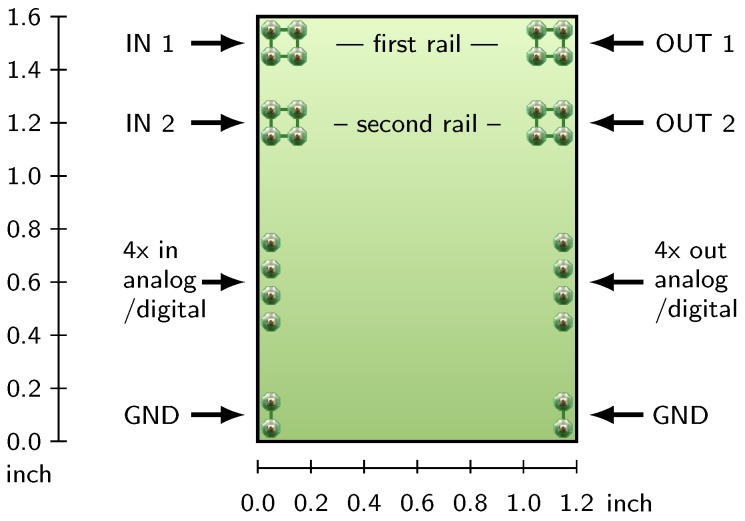
Standard module layout to hold one power management function, measuring 1.2${}^{\prime \prime }$ × 1.6${}^{\prime \prime }$.

The standardized module is supposed to hold either a PMIC or an energy storage. Along the PMIC all necessary passive components, such as resistors, capacitors and inductors, should be integrated on a module. We analyzed commercially available PMIC regarding their space constraints and developed a standardized layout which is shown in Figure 4. The module measures 30.48 mm × 40.64 mm (1.2${}^{\prime \prime }$ × 1.6${}^{\prime \prime }$) which is large enough to lay out each PMIC adequately. The very basic concept and an early list of developed modules are reported by the authors in [31]. The design files are available as open-hardware under [32]. The purpose of the pins of each module is explained next:

**Two power rails** on every module are reserved for the transfer of energy (IN1, IN2, OUT1, OUT2). A four-pin connection with 0.1${}^{\prime \prime }$-pin headers reduces the contact resistance and results in a reliable mechanical connection, as verified in Section 4.1.

**Control signals** are analog or digital signals which are located on the left and right side of the module. To connect the control signals between the modules, regular 0.1${}^{\prime \prime }$-jumper wires are used. Depending on the layout, some input signals can be directly controlled by jumpers at the module to set the mode of operation and thereby use the module as a stand-alone device.

**Two ground connections** are provided for every module to increase the reliability. As there is no need for galvanic isolation between components in a µPM, all grounds are connected together.

### 3.4. Base Board

**Figure 5:**
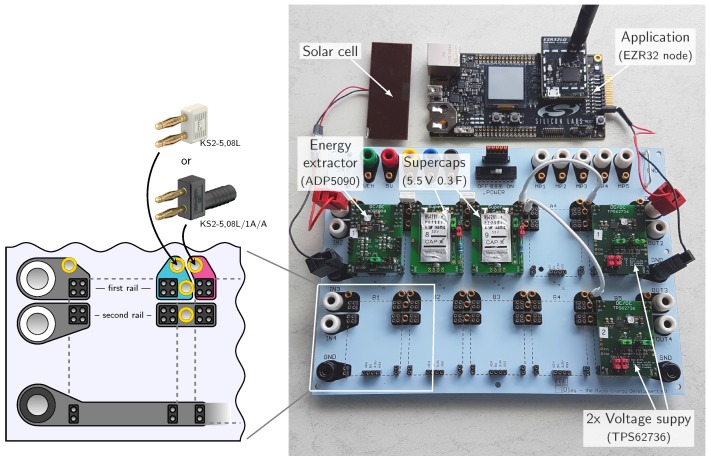
Photo of Medlay with an example setup. Energy from a solar cell is extracted by the ADP5090 and stored on a supercapacitor array. A regulated voltage (3.3 V and 3.6 V) is provided by two TPS62736.

Figure 5 shows an example setup of Medlay with the base board in light blue. Energy from a solar cell is extracted by the ADP5090 from Analog Devices and stored on two supercapacitor modules (Cap-XX, 0.3 F). A regulated voltage (3.3 V) for the application (a Silicon Labs EZR32 starter board) is provided by a TPS62736 dc-dc converter from Texas Instruments. The output of the 2nd TPS62736 providing 3.6 V is not connected and exemplary shows how to connect additional circuitry.

The baseboard is designed to hold up to 10 modules in two rows. Banana plugs on the left and right edge allow for connecting harvesters, loads and laboratory equipment. The base board has conducting paths which connect the modules to each other as illustrated in the bottom left corner of the figure. In between two neighboring modules 4 banana sockets (⌀ 2 mm) are provided. The first power rail is interrupted, which is shown in turquoise-pink, whereas the second rail is connected from module to module. The interruption in the first rail becomes useful when disabling circuit parts temporarily or when currents shall be measured between modules. The connection is restored using the shunt KS2-5,08L from *Multi-Contact*. The second rail is connected trough, as it is unlikely that signals of the second rail interfere among each other. Banana cables (⌀ 2 mm) can be used to connect any of the remaining 2 mm banana plugs. Five adapter points are additionally provided at the top right of the base board, where five 2 mm and 4 mm plugs are connected pairwise.

### 3.5. Guidelines for the Modular Composition

The following guidelines are helpful, when a system is assembled with Medlay:

1. Organize your system concept or µPM circuit according to the structure shown in Figure 2. Identify the functions *energy extraction*, *storage interaction* and *voltage supply*.
2. Choose suitable modules for the power functions and energy storages from the kit. Design missing modules according to the specification given in Section 3.3.
3. Connect energy harvesters to the input banana plugs. This allows harvesters to be set up properly in the environment (e.g., the optimal orientation of a PV panel).
4. Connect the supply pins of the load (e.g., a microcontroller or sensor board) to the output banana plugs or use the dummy load module for testing (see [33]).
5. Place dc-dc converters for the primary energy extraction and voltage supply in the first row of the base board.
6. Place additional functions like a second harvester, battery backup and switches for secondary loads in the second row.

## 4. Results

### 4.1. Parasitic Effects of Interconnections

One could argue that tearing apart the power management into separate modules and increasing the distance between components may influence the power management. In this case, the increase of resistance, capacitance and inductance are the relevant sources of error. To resolve these doubts we conducted measurements on the PCB (printed circuit board) traces and interconnections. Table 3 summarizes the results. The contact resistance was measured under a test current of 1 A and by applying a four wire measurement. Furthermore we measured the isolation resistance and the capacitance between traces.

### 4.2. Versatility Evaluation w.r.t. Base Board Sizes

For a first rough estimate, how versatile the system is, we calculate how many module combinations exist for different base board sizes. As shown in Table 2 there are numerous thinkable modules. As there is no maximum number of power components, we need an abstraction. Our solution is to summarize the modules into four classes, which only differ in the number of power input and power output ports. The four classes are: siso, sido, diso, dido where we use the abbreviations: s = single, d = double, i = input, o = output.

In the following, let *m* be the number of different classes available which equals to m=4 in our case. If a base board with one row and *c* columns is fully populated, the resulting number of configurations is $m^{c}$. To consider unpopulated sockets in the next step, we need to add all lower-order systems (1 to c−1) too. As $m^{c}$ is a geometric sequence we use the formula of the sum of the first *c* terms of a geometric series. The total number of states *s* is then given by Equation (1). If a second row is added, Equation (2) gives the number of all possible states. Here, equivalent systems e.g., due to swapping two identical rows, are considered by the Gaussian sum formula. Table 4 shows in the top half the calculated values for Equations (1) and (2). With that calculation it must be noted, that neither actual wiring nor connection limitations of the base board are taken into account.

(1) $$\begin{array}{c} s_{1\times c}=\sum_{i=1}^{c}m^{i}=\frac{1-m^{c+1}}{1-m} \end{array}$$

(2) $$\begin{array}{c} s_{2\times c}=\frac{s_{1\times c}\;\cdot \;\left(s_{1\times c}+1\right)}{2} \end{array}$$

For a deeper investigation, we represent the numerous configurations of the testbed by attributed graphs. In general, graphs can be used to represent states of sets of objects with relations between them. As there are so many configurations (and consequently so many graphs) a rule system is needed which is called *graph grammar*. This very compact notation contains all valid graphs and thus delivers a valid description of all architectures which can be built with Medlay. As a counter-example, non-valid architectures contains modules with three inputs.

**Figure 6:**
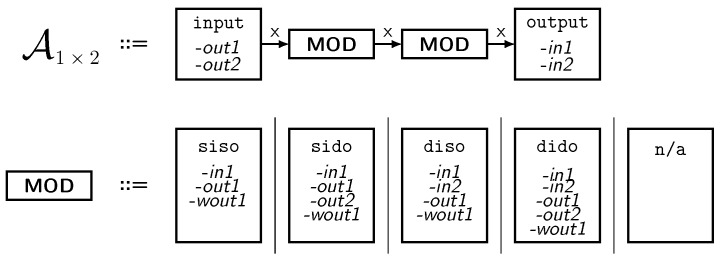
Top: start graph representing an unspecified 1×2 base board. Bottom: example of a graph transformation rule to replace unspecified modules by individual modules (siso to n/a).

The graph grammar consists of graph transformation rules in which the nodes and vertices of the graph are modified according to a fixed pattern. Using software, the transformation rules can be applied automatically to explore all possible configurations of a system. Figure 6 (top) shows a start graph A (axiom) of the grammar, which represents an unspecified 1×2 base board. Here, each top class MOD is a non-terminal symbol representing one valid module. The board is then populated with modules by applying the transformation rule shown in Figure 6 (bottom). To model the situation where a socket remains unpopulated, a fifth class called n/a (not available) is introduced. The grammar involves attributes, namely *in1*, *in2*, *out1*, *out2* and *wout1*. The attribute *wout1* is the wire output for rail 1 as seen in turquoise in Figure 5.

Rules which represent wires and jumpers are applied in the next step based on the generated class attributes. Due to brevity, the full grammar cannot be shown here, but is available as supplemental material. We decided to use the tool *Groove* [34] to model and solve the graph transformation problem. The tool automatically detects isomorphism (two setups which are electrically identical) and counts the distinct number of configurations.

The simulation results are shown in the lower half of Table 4. It is remarkable, how strong the number of buildable systems increases. Unfortunately, we were not able to complete the simulation for the base boards 2×4 and 2×5. The calculation on a Intel Xeon machine (16 core @ 3 GHz) with 64 GB RAM ran into a out-of-memory error. Nevertheless it becomes clear, that over 250,000 configurations with the 2×5 board are sufficient in reality.

### 4.3. Applicability Demonstration of the Modular Platform

**Figure 7:**
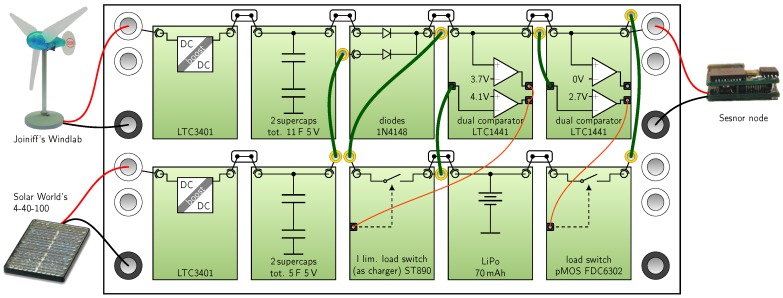
Power management of *Ambimax* [18] rebuilt with Medlay.

**Figure 8:**
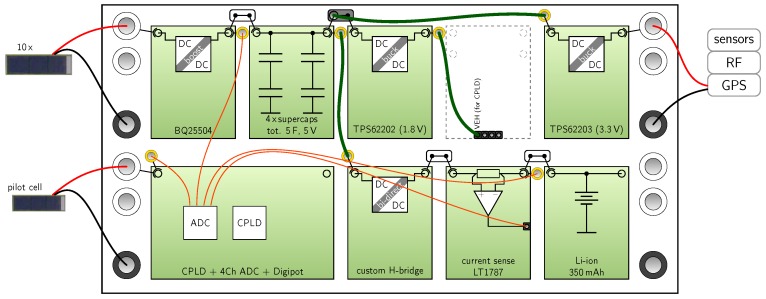
Power management of *Trio* [19] rebuilt with Medlay.

**Figure 9:**
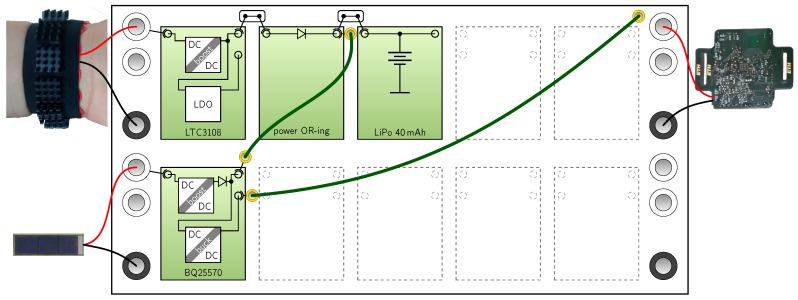
Power management of *InfiniTime* [23] rebuilt with Medlay.

To demonstrate possible applications, we show how three power managements from literature could be rebuilt with our modular kit. We selected [18] (Ambimax), [19] (Trio) and [23] (InfiniTime). The first two systems are selected because they implement a hybrid storage and custom control circuitry. The third system is chosen because it utilizes power converters with additional circuitry (LDO and buck converter). Figure 7, Figure 8 and Figure 9 show the setup with the harvesters and loads of the original work. We observed that extracting the circuit data from the original publication was time-consuming due to their non-standardized presentation. Whereas in our extraction results the functional blocks are clearly visible. In Figure 7 multi-harvesting is realized using a wind harvester and a pv cell. For each harvester a reservoir capacitor array (RCA) is realized as one module. For modules that can be reused, we suggest designing two identical comparator modules which are configured with different reference voltages by resistors. In Figure 8 ten PV cells are connected to the first row, where the pilot cell is connected to the second row connected to the CPLD. For the 100-pin CPLD we recommend a double module. To monitor three internal voltages and the battery current, sensor wires shown in orange are connected to the CPLD. In Figure 9 the BQ25570 already includes one power or-ing diode and a buck converter for the voltage supply, which makes the setup quite compact. The LDO of the LTC3108 is not used in this setup.

Although we did not recreate the systems entirely, we build complex systems with similar modules for comparable WSN applications. From that we confirm that there are no noticeable design restrictions due to the high number of possibilities (see Section 4.2).

## 5. Discussion

The majority of the test platforms [25,27,28,29] presented in Section 2.2 are not modular in the physical aspect. Especially the commercial solutions are very limited as the companies only compare PMIC in their portfolio. They integrate the whole circuitry on one PCB which is a robust and cost-effective solution but totally inflexible. Due to numerous jumpers, the energy flow is not clearly traceable on the board.

Modular in the physical aspect are the concepts [24,26,30]. As confirmed in Section 4.1 the physical modularity has negligible parasitic effects. However, an issue in [24] is that power converters are fixed to a storage or a load. Further, the multiplexer and the EEPROM introduce an overhead that complicates the power management structure. The solution presented in [26] offers a very flexible usage, but there is no assertion of the 32 I/O lines. Soldering in the connection matrix is involved and thus the setup may end up a bit unorganized. Another drawback is the size of the modules (16×10 cm)—a one meter wide system would result, if 10 modules are connected together. The EH Platform from Future Electronics [30] was a promising solution but is not available any longer. As the baseboard has dedicated ports (one harvester, two dc-dc converters, one storage and one load module) the structure is limited from the outset. Further, the rigid build of relays and current sensing cannot be adapted to user needs.

Our approach called Medlay overcomes all mentioned issues regarding versatility, clarity and size as compared in Table 5. **Versatility:** All enhanced concepts like multi-source harvesting or a hybrid storage can be realized with Medlay. All functions such as power converters and energy storages are fully exchangeable. Furthermore, the structure of a µPM is freely adaptable using 2 mm banana connectors. To enable others to utilize the development effort already invested, the platform is provided as open-hardware under [32]. **Clarity:** The individual µPM functions become evident by following the proposed interface, as power and status pins are clearly separated. The uniform interface results in a clear layout with the flow of energy from left to right due to fixed input and output positions. **Size:** The modules are comparably small (see Table 5) which makes the total setup compact. A system built up with Medlay can be deployed directly in a real application, as it fits into an IP66 housing (Hammond 1554).

## 6. Conclusions

The variety of PMIC and energy storages available shows that there is a need for a general physical structure. Although power functions such as energy extraction reoccur, only a uniform interface makes testing and exchanging components feasible. To approach that, we analyzed commercially available PMIC regarding their layout constraints and number of inputs and outputs. We conclude that two inputs and two outputs for power are sufficient and proposed an according interface. Based on that, we designed the open-hardware platform called Medlay consisting of a base board and uniform modules. The name Medlay derives from *medley* (of songs) and *layout*, as individual power management functions can be composed freely to form new layouts.

We studied the versatility limits of the testbed and thereby followed a theoretical and a practical approach: by using a layout graph grammar the number of individual setups of Medlay was verified to over 250,000. Additionally, the recreation of three state-of-the-art systems confirmed this flexibility. The measured parasitic effects of interconnections are negligible and consequently a setup with Medlay exactly represents a system on one pcb.

The kit helps to understand complex micro-power systems and supports the development of power management in an early design phase. The ready-to-use modules assist the user to build a working prototype which makes the kit an excellent tool for teaching students in an embedded systems course. Medlay is now fully developed and will be used in our future research projects as a tool during development. Applications are (1) a solar-powered smart brick in the track bed of a tram line to monitor wear and (2) an energy-aware gas monitoring system with dynamic duty-cycling.

## Figures and Tables

**Table 1:** State-of-the-art power management systems structured by their key components.

Ref.	Energy Generator(s)	Energy Extraction	Energy Supervisor	Storage Interaction	Energy Storage(s)	Voltage Supply
[18]	solar 0.3 W + wind 0.5 W	LTC3401, pilot elem. and LTC1440 comp.	or-ing diodes, LTC1441	curr. lim. switch ST890	supercaps 11 F, 5 F and LiPo	pMOS selects energy source
[19]	10 mini PV cells 17.9 mW	BQ25504, pilot cell, controlled by CPLD	CPLD and four ADCs	custom H-bridge buck/boost	supercap 5 F and 350 mAh Li-ion battery	buck conv. TPS6220x
[20]	solar 5 W	TPS43000 (sepic)	PIC µC and INA213	TPS43000 (bi-dir. buck and boost)	supercap 3× 350 F 2.7 V and 680 mAh battery	connected to 3.3 V bus
[21]	TEG (250 mV at 4 K)	S-882Z18 (charge pump) + TPS61020	µC for SoC detection	OV incl. in TPS61020	4x supercaps, 2.5 F, 5 V in total	TPS61220
[22]	vibration + solar	LTC3401 and LTC3632	passive: or-ing diodes	resistor divider, pMOS and diode	supercap 25 mF 5 V + 130 mAh Li battery	buck-boost (*type not given*)
[23]	TEG + solar	LTC3108 and BQ25570	passive: or-ing diodes	UV and OV part of dc-dc conv.	LiPo 40 mAh 3.7 V	buck of BQ25570

**Table 2:** Analysis of the pin assignments of commercially available power management components regarding power signals (IN/OUT) and control signals (A/D). A star indicates that a function is not available on every component.

Type	IN 1	IN 2	OUT 1	OUT 2	A/D In	A/D Out
DC-DC (e.g., ADP5090, BQ25570)	harvester pos. input	battery backup input *	intermediate storage out	regulated output *	chip enable, force MPP *	power good *
AC-DC (e.g., LTC3109)	harvester pos. input	harvester neg. input	intermediate storage out	regulated output *	chip enable	power good *
Energy storage (e.g., supercap)	storage pos. pin	-	(short to IN1)	-	balancing tap *	3-wire sense
Switch matrix (e.g., ADG888)	switches in	switches in*	switches out	switches out *	control	power good *

**Table 3:** Overview of parasitic effects of the modular micro-power management.

Quantity	Result
Contact resistance (module to board)	5 ± 0.1 mΩ
2 mm banana wire and 2 banana plugs	7.8± 0.1 mΩ
Isolation resistance (IN1 to GND)	>100 GΩ
Capacitance (IN1 to GND)	<1 pF
Capacitance (IN1 to IN2)	<1 pF

**Table 4:** Number of buildable systems: Top half: rough calculation of module combinations (without wiring). Lower half: Groove simulation results by considering module connections.

Scope (Method)	Cols. *c*	1	2	3	4	5
modules only	1 row	5	21	85	341	1365
(Equations (1) and (2))	2 rows	15	231	3655	58,311	932,295
with wires	1 row	5	18	90	1094	82,879
(Groove)	2 rows	24	2550	244,009	>250,000	

**Table 5:** Overview of evaluation systems for energy harvesting and micro-power management.

Name and Publication	Physical Modularity	Number of Harvesters	Energy Path Routing	Versatility of Power Converters	Versatility of Energy Storages
Weddell et al. [24]	max. 6 modules, ≅ 40 mm×55 mm	use 2 of 2 (pv, vibr.)	selected by analog MUX	converter fixed to harv. or storage	supercap and Li-battery
Bader et al. [25]	no, one PCB (size not given)	use 1 of 1 (pv)	fixed, interrupt by jumpers	fixed (LTC3105 and MAX17710)	supercap and thin-film bat.
Nagel et al. [26]	2 to 5 modules, 100 mm×160 mm	use 1 of 1(rf)	free assignment to 32-pin bus	designed for one per card	designed for one per card
ADP5091 Demo [27]	no, one PCB 120 mm×120 mm	use 1 of 2 (pv, teg)	fixed, interrupt by jumpers	ADP5091 (fixed)	fixed, one LiPo (240 mAh)
WE EH to go [28]	no, one PCB 94 mm×125 mm	use 1 of 3 (pv, teg, ext.)	fixed, interrupt by jumpers	select between 4 dc-dc by jumpers	MLCC array (15 × 100 μF)
WE Gleanergy [29]	no, one PCB 94 mm×125 mm	use 1 of 3 (pv, teg, ext.)	fixed, interrupt by jumpers	select between 4 dc-dc by jumpers	MLCC array and supercaps
Future Electronics EH [30]	max. 4 slave modules ≅ 40 mm×70 mm	use 1 of 1 (pv)	fixed, interrupted by 4 relays	select between 2 dc-dc modules	1 supercap module (2 F, 5.5 V)
**this work**	max. 10 modules, 30 mm×40 mm	use 2 of 2 (arbitrary)	base board and 2 mm banana plugs	fully replaceable, >10 types available	fully replaceable, >5 types avbl.

## Abbreviations

The following abbreviations are used in this manuscript:

ADC	analog-to-digital converter
CCCV	constant-current, constant-voltage (charging scheme)
CPLD	complex programmable logic device
EEPROM	electrically erasable programmable read-only memory
EH	energy harvesting
FPGA	field programmable gate array
FOCV	fractional open-circuit voltage (method)
I/O	input/output
LDO	low-dropout regulator
MLCC	multi-layer ceramic capacitor
MPP	maximum power point
PCB	printed circuit board
PMIC	power management integrated circuit
PV	photovoltaic (generator)
TEG	thermoelectric generator
µPM	micro-power management
WSN	wireless sensor nodes
